# Confirmatory Factor Analysis of the Motor Unified Parkinson's Disease Rating Scale

**DOI:** 10.1155/2012/719167

**Published:** 2012-10-22

**Authors:** Stefanie D. Vassar, Yvette M. Bordelon, Ron D. Hays, Natalie Diaz, Rebecca Rausch, Cherry Mao, Barbara G. Vickrey

**Affiliations:** ^1^UCLA Department of Neurology, Box 951769, C109 RNRC, Los Angeles, CA 90095-1769, USA; ^2^VA Greater Los Angeles Healthcare System, Parkinson's Disease Research, Education and Clinical Center, Los Angeles, CA 90073, USA; ^3^UCLA Division of General Internal Medicine and Health Services Research, Department of Medicine, Los Angeles, CA 90095, USA; ^4^UCLA School of Public Health, Los Angeles, CA 90095-1772, USA; ^5^RAND, Santa Monica, CA 90407, USA; ^6^Department of Neurology, Harbor-UCLA Medical Center, Torrance, CA 90502, USA

## Abstract

The motor examination section of the unified Parkinson's disease rating scale (UPDRS) is widely used in research but few studies have examined whether subscales exist that tap relatively distinct motor abnormalities. We analyzed data from 193 persons enrolled in a population-based study in Central California. Patients were examined after overnight PD medication washout (“OFF” state) and approximately one hour after taking medication (“ON” state). We performed confirmatory factor analysis of the UPDRS for OFF and ON state examinations; correlations, reliability, and relative validity of resulting subscales were evaluated. A model with five factors (gait/posture, tremor, rigidity, bradykinesia affecting the left extremities, bradykinesia affecting the right extremities) fit the data well, with similar results for OFF and ON states. Internal consistency reliability coefficients were 0.90 or higher for all subscales. The gait/posture subscale most strongly discriminated across levels of patient reported PD symptom severity and of how PD affects them on a daily basis. Compared to the right sided bradykinesia subscale, the left sided bradykinesia subscale had higher discrimination across levels of self-reported PD symptom severity and functional impairment. This supports motor UPDRS containing multiple subscales that can be analyzed separately and provide information distinct from the total score that may be useful in clinical studies.

## 1. Introduction

The motor examination section (Part III) of the unified Parkinson's disease rating scale (UPDRS) is the most widely used measure to assess motor symptoms and signs in Parkinson's disease (PD) [1]. The motor section is the only component of the UPDRS where items are scored by the physician rather than by patient self-report. An overall measure of motor abnormalities may not discern beneficial effects of treatments that might target certain motor components, or enable identification of subsets of patients with different motor examination profiles and prognoses. 

A previous study reported an exploratory factor analysis of the factor structure of the motor UPDRS in a sample of patients with idiopathic PD in the ON state (taking their usual medications) [2]. Six factors were identified; axial functioning and gait, rest tremor, rigidity, bradykinesia affecting the left extremities, bradykinesia affecting the right extremities, and postural tremor. However, this prior study employed principal component analysis, and assumed continuous data. The motor UPDRS consists of five-category ordinal items scored 0–4. A followup study performed a confirmatory factor analysis in the OFF state (as part of regular care visits throughout the day) in an independent sample and found similar item loadings for the 6 factors obtained from initial exploratory factor analysis in the ON state [3]. 

All previous evaluations of the factor structure of the Motor UPDRS have been in separate samples of PD patients in either the ON or OFF states [2, 3] or in a sample containing patients with mixed medication states [4]; therefore, differences in factor structure between medication states were not assessed. 

The present study re-examines the previously defined factor structure of the items of the motor UPDRS using categorical confirmatory factor analysis in both ON and OFF states in the same group of patients, accounting, for the ordinal distributions of the motor UPDRS items.

## 2. Methods

### 2.1. Sample

The Parkinson's, Environment, and Gene (PEG) study enrolled 371 subjects between 1998 and 2006 who had been diagnosed with PD within the prior three years [5, 6]. Between June 2007 and June 2009, 254 of the original cohort were eligible, consented, and participated in a followup study assessing the progression of disease. Patients were examined by a movement disorder specialist and completed in-person and telephone interviews [7]. The UPDRS was administered in both the OFF medication state (patients withholding all PD medications for at least 12 hours prior to assessment) and the ON medication state (one hour after taking their usual morning PD medications that same day). Subjects without both an OFF and ON exam were excluded from this analysis. Reasons for not completing both exams included, not being able to withhold morning medications (*n* = 16; only ON exam performed), no PD medications prescribed (*n* = 12; only OFF exam performed), did not bring medications to the exam (*N* = 3; only OFF exam performed), or no reason recorded (*n* = 6). An additional 24 participants could not undergo an in-person exam, yielding an analytic sample of 193. 

The UCLA IRB (#G06-07-055) approved all study procedures, and all subjects provided informed consent.

### 2.2. Measures

The motor UPDRS exam is the summation of 27 physician-rated items each scored on a 0 (no impairment) to 4 (severe impairment) categorical scale. Items are included assess motor abnormalities such as rest tremor, action tremor, rigidity, bradykinesia, gait and posture, and facial masking. The total motor UPDRS exam score ranges from 0 to 108.

Self-ratings of the effects of PD on day-to-day activities and PD symptom severity were assessed during telephone interviews administered by trained research assistants. Responses from 23 patients were excluded because the self-reported ratings were administered more than 30 days after the UPDRS exams. Participants were asked to indicate one choice that “best describes how your Parkinson's disease has affected your day-to-day activities in the last month:” (1) no difficulties, (2) mild difficulties, (3) moderate difficulties, (4) high levels of difficulties, or (5) extreme difficulties. Each response option was followed by a detailed example. This single item was developed specifically for PD based on interviews with PD specialist clinicians, patients, and caregivers, as well as on a literature review. Construct validity of the item was supported by its associations with depression, cognition, and PD severity in a community based PD sample [8]. Participants were also asked to rate the severity of their PD symptoms in the past 6 months as (1) no symptoms, (2) mild symptoms, (3) moderate symptoms, and (4) severe symptoms. 

### 2.3. Data Analyses

Categorical confirmatory factor analysis was performed on the 27 items of the motor UPDRS to evaluate factors identified previously in PD patients in both ON and OFF states [2, 3]. Models were estimated using weighted least square parameter estimates using a diagonal weight matrix with robust standard errors using MPLUS version 6 software (Los Angeles, CA) [9]. Analyses were performed using OFF state total motor UPDRS scores to capture untreated motor symptoms. Model fit was evaluated using three goodness-of-fit indices including the comparative fit index (CFI), the Tucker-Lewis Index (TLI), and the root mean squared error of approximation (RMSEA). In general, a CFI above 0.95 [10], a TLI above 0.95, and a RMSEA value lower than 0.06 signify good model fit [11]. Finding the optimum model is an exploratory process, but is a common procedure when the original model does not have the best possible fit [12]. In the initial model parameter from the Stebbins model were fixed and all correlations were set free. Then, using modification indices and conceptual acceptability a series of models were fit fixing correlations between items and correlations between subscales. Once a final model was determined, the same model was performed for the ON state UPDRS items, and results were compared.

Scales were calculated by taking the mean of the final set of items from each factor in the confirmatory factor analysis, transformed linearly to have a 0 to 100 possible range, where 0 is the best state. For each scale, the mean score, standard deviation, and observed minimum and maximum were calculated. Internal consistency reliability was estimated using Cronbach's alpha [13]. Subscales with a Cronbach's alpha of 0.70 or greater are considered adequate for group comparisons while scales with reliabilities of 0.90 or above are sufficient for individual applications [14].

Relationships between the total motor UPRDS score and newly developed scale scores in the ON and OFF states were assessed using product-moment correlations. We classified correlations greater than 0.50 as large, 0.30–0.50 as moderate and 0.10–0.29 as small [15]. To eliminate overlap between the total motor UPDRS scores and the scales, items for a particular scale were excluded from the total motor UPDRS score prior to computing the product-moment correlation.

Relative validity evaluates the extent to which two or more scores are associated with an external criterion as hypothesized [16]. We evaluated the relative validity of the total motor UPDRS score and the scales relative to two external criteria; the single item self-rating of the impact of PD on day-to-day activities, and the single item self-rating of severity of PD symptoms. Mean scores on the motor UPDRS total and subscales were compared across response levels of each item. One-way ANOVA F-statistics were computed for each scale, and the significance of differences between pairs of groups was estimated using Duncan's multiple range test. We report relative validity as F-statistics; the ratio of a scale's F-statistic to that of a reference scale (the scale with the smallest F-statistic). Therefore, the scale with the highest F-ratio is the most sensitive to differences across categories of the external criteria variable [17]. 

## 3. Results

Participants had an average age of 73 years, 41% were female and 79% were non-Hispanic white. The average duration of PD diagnosis was 5 years, and 72% were Hoehn & Yahr stage (in OFF state) 2.5 or lower. Nearly 35% of participants were taking levodopa only, 56% were taking levodopa with another medication (i.e., amantadine, trihexyphenidyl and/or entacapone), and only 9% were taking a dopamine agonist without levodopa (Table 1).

### 3.1. Confirmatory Factor Analysis

**Figure 1:**
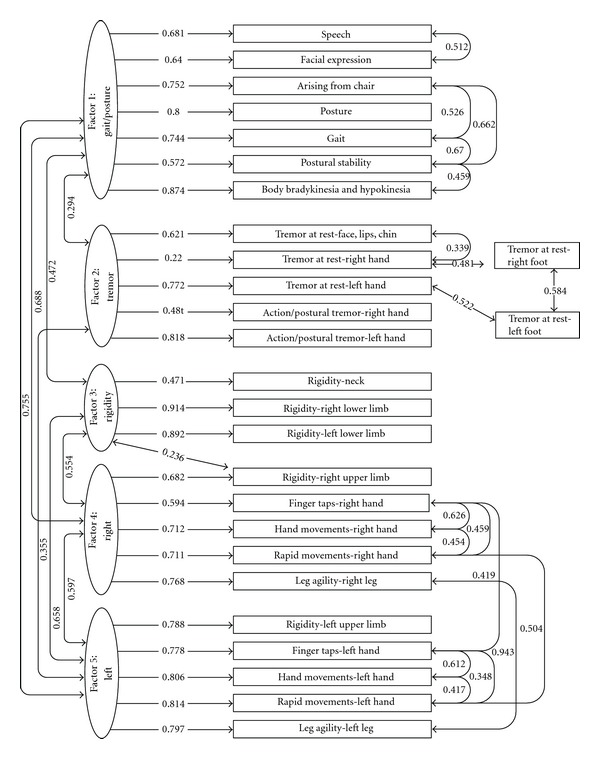
Confirmatory factor analysis results OFF medication. Single headed arrows indicate factor loadings, which double headed arrows indicate residual correlations.

We started with the 6-factor model previously reported [2, 3], but after fitting eight sequential models, we found that a five factor model fit the data better: CFI = 0.962, TLI = 0.955, RMSEA = 0.059 (Figure 1). Factor loadings ranged from 0.22 to 0.91 (median = 0.75; standard deviation = 0.15). This model included 19 unique correlations between pairs of items, denoted as residual correlations in Figure 1.

Items measuring speech, facial expression, arising from a chair, posture, gait, postural instability, and body bradykinesia comprised Factor 1. Factor 2 included the three rest tremor measures and both postural tremor measures. Foot tremor scores did not meet criteria to be included directly into Factor 2, but correlated best with ipsilateral hand tremor measures. Factor 3 is made up of rigidity measures (neck, right, and left lower limbs). Factors 4 and 5 include measures of right and left bradykinesia, respectively. 

When this same 5-factor model was estimated for UPDRS items in the ON state, similar fit (CFI = 0.963, TLI = 0.956, RMSEA = 0.060) and factor loadings were obtained to that found for the OFF state (Table 2). 

Internal consistency reliability was 0.90 or higher for the five subscales formed from the items loading on the five factors (Table 3). There were floor effects for the rigidity subscale in the ON state (53% of the sample scored the possible minimum = least rigidity) and the tremor subscale in the ON state (38% of the sample scored the possible minimum). These floor effects were greatly reduced in the same scales in the OFF state (28% of the sample scored the possible minimum on the rigidity subscale, and 14% scored the possible minimum on the tremor subscale). 

### 3.2. Correlations of Total Motor UPDRS Score with the Five Scale Scores

Table 4 shows the correlations of the total motor UPDRS score with the five subscales for both OFF and ON states, corrected for item overlap. The gait/posture, rigidity, and right and left bradykinesia subscales had moderate to high correlations with the total motor UPRDS score both in the OFF state (*r* = 0.45–0.65) and ON state (*r* = 0.43–0.70). The tremor subscale has the lowest association with the total UPDRS (*r* = 0.19 OFF state, *r* = 0.27 ON state). There were small correlations between the tremor subscale and the other subscales, both OFF medication (*r* ≤ 0.17) and ON medication (*r* ≤ 0.23). 

### 3.3. Relative Validity

*How PD affects you on a day-to-day basis*: Sixteen percent of participants reported no difficulties, 56% reported mild difficulties, 22% reported moderate difficulties, and 6% reported high levels or extreme difficulties. Due to few responses for high levels of difficulties and extreme levels of difficulties, these two levels were combined. The level of discrimination across the four categories was high for the total motor UPDRS (relative validity = 35.43 OFF state and 37.43 ON state). Of the 5 subscales, gait/posture subscale had highest relative validity (relative validity = 37.95 ON state and 37.38 OFF state). The tremor subscale had the lowest relative validity of the five subscales (relative validity = 2.26 OFF state and 1.00 ON state). Right and left subscales had similar discrimination in the OFF state, while the left subscale had higher relative validity compared to the right subscale in the ON state. (Table 5(a)).

*PD symptoms severity rating*: four patients reported no PD symptoms, 90 reported mild symptoms, 88 reported moderate symptoms, and 10 reported severe symptoms. The total motor UPDRS had high discrimination across the four PD symptom severity categories (relative validity = 5.56 OFF medication and 6.47 ON medication).The gait/posture subscale OFF medication, and left bradykinesia subscale ON medication had higher relative validity than the other subscales (relative validity = 5.55 and 5.82, resp.). The tremor subscale had the lowest relative validity (relative validity = 1.00 OFF medication and 1.79 ON medication). (Table 5(b)). 

## 4. Discussion

 This study evaluated the six-factor solution of the motor section of the UPDRS identified in prior studies. We found that all tremor items fit on the same factor rather than being divided into rest tremor and postural tremor. The other four factors we identified were consistent with prior work [2, 3]. Similar to Stebbins et al., a 7-item gait/posture factor was identified that includes speech, facial expression, arising from a chair, posture, gait, postural instability, and body bradykinesia. However, all tremor items in our model loaded together on one factor, as opposed to the analysis by Stebbins et al., where the two postural tremor items fell into a separate factor from the rest tremor factor. The foot tremor items that were included in Stebbins' rest tremor factor did not meet load on the gait/posture factor in our analysis, but are correlated with the hand tremor items. Analogous to Stebbins et al., we found the rigidity measures formed a factor, as well as two factors measuring right and left bradykinesia. The right and left upper limb rigidity items loaded on the right and left bradykinesia factors, rather than on the rigidity factors that was reported in the Stebbins model.

The differences we found could be due to variation caused in a new sample. It is also possible that differences in the assumptions of the analyses contributed to the differences. That is, we use an estimation that accounted for the ordinal nature of the motor UPDRS items. 

The low correlations of the tremor scale with the other subscales in both ON and OFF medication states suggests that the tremor subscale is measuring distinct aspects of PD impairment. Stebbins et al. found similar low correlations when the same items were split into the rest tremor and postural tremor factors in both medication states. The low correlations of tremor with other symptoms may reflect the relative refractory improvement of tremor to medications compared to rigidity and bradykinesia. In addition, tremor predominant PD cases present in the population would contribute to this dissociation as well and be an indicator of the lack of relationship between higher level of tremor and greater PD impairment. The low correlation of the tremor subscale with other symptom scales also supports previous suggestions that tremor in PD may have different pathophysiological mechanisms as compared to the other motor symptoms of PD [18–20].

Analyses revealed that the gait and balance subscales had a larger impact on subjective reporting of PD symptom severity and functional impairment due to PD. Conversely, tremor had little contribution to these ratings. This suggests that the features of PD that have the greatest functional impact are those that impair independent mobility. Mobility issues become quite problematic as the disease progresses, given the need for acceleration of the use of assist devices (from cane to walker to wheelchair). 

Similar to Stebbins et al., side-sensitive bradykinesia was represented in the right and left factors. The right subscales had lower relative validity than the left subscales for both self-reported PD symptom severity and functional impairment. This could be due to greater difficulties with involvement of the dominant (right) versus nondominant side of the body, or to increased verbal problems reportedly associated with greater right-side involvement [21]. Also, depression is common in PD, and there is some literature to suggest an association between depression and laterality of motor symptoms in PD [22, 23]. Further research with these UPDRS subscales, subjective self-report PD severity, and impairment and their association with hand dominance, cognition, and mood is indicated. 

The factor structures were very similar for the ON state and OFF state. Because the OFF state captures untreated motor symptoms and covers a wider range of disease severity, the resulting scales may be generalizable to groups with different of ranges disease severity. In addition, all subscales had very high internal consistency reliability, exceeding or equaling 0.90.

Regarding potential limitations, a low subject-to-variable ratio can lead to higher standard errors. However, the parameter estimates and correlations used in our models were all significant, and our subject-to-variable ratio of 7 : 1 exceeded a recommended minimum of 5 : 1 [24, 25].

These findings support prior work that identified an underlying structure of motor UPDRS examination and support the use of the resulting subscales in clinical and research settings to assess separate aspects of motor abnormalities of PD to enable determination of differential treatment effects or prognosis. 

While the newer version of the scale, the MDS-UPDRS, is gaining wider use, there is a large overlap of the items from the original UPDRS to the MD-UPDRS, and mapping of ratings between the two scales exist [26]. Evaluations of the two scales show extremely high correlations between the motor sections of the MDS-UPDRS and UPDRS [26, 27]. These findings could be a starting point for exploration of possible subscales within the MDS-UPDRS. 

## Figures and Tables

**Table 1:** Clinical and sociodemographic characteristics (*n* = 193).

	*N* (%) unless specified
Age in years: mean (SD)	72.4 (9.2)
Female	79 (41)
Ethnicity	
White or European American	152 (79)
Latino or Hispanic	30 (16)
Other*	11 (6)
Highest level of education	
Did not finish grade school (grades 1–8)	10 (5)
Finished grade school but no high school diploma	17 (9)
High school diploma	73 (38)
Technical or trade school diploma	21 (11)
College diploma	34 (18)
Graduate school diploma	28 (15)
Cannot remember/Refused/Do not know/Other	10 (5)
Marital status (*n* = 190)	
Never married	6 (3)
Married	145 (76)
Divorced/Separated	15 (8)
Widowed	24 (13)
Work status	
Employed	35 (18)
Retired	143 (74)
Other: unemployed (5), disabled (8), emeritus (1), housewife (1), temp work (1)	15 (8)
Mean duration of PD diagnosis, years (SD)	5.2 (2.3)
Modified Hoehn and Yahr Stage (OFF state)	
Stage 1: Unilateral disease	10 (5)
Stage 1.5: Unilateral plus axial involvement	9 (5)
Stage 2: Bilateral disease, without impairment of balance	64 (34)
Stage 2.5: Mild bilateral disease with recovery on pull test	54 (28)
Stage 3: Mild to moderate bilateral disease; some postural instability; physically independent	34 (18)
Stage 4: Severe disability; still able to walk or stand unassisted	12 (6)
Stage 5: Wheelchair bound or bedridden unless aided	7 (4)
Medication status**	
Levodopa only	67 (35)
Levodopa and any other medication	108 (56)
Dopamine agonists without Levodopa	17 (9)
How Parkinson's disease affects you on a day-to-day basis?***	
No difficulties	27 (16)
Mild difficulties	94 (55)
Moderate difficulties	39 (23)
High levels or extreme difficulties	10 (6)
During the past 6 months, how would you rate the severity of your Parkinson's disease symptoms? ***	
No symptoms	4 (2)
Mild symptoms	79 (47)
Moderate symptoms	79 (47)
Severe symptoms	8 (5)

*Other race includes African America, Native American, or Asian or Pacific Islander.

**One subject excluded because they were on MAO-B inhibitors alone.

***Subjects with self-rated reports within 30 days of motor UPDRS exam (*n* = 170).

**Table 2:** Factor loadings for confirmatory factor analysis off and on Parkinson disease medications.

	OFF medication	ON medication
	Factor loadings	Factor loadings
Tests of model fit		
CFI	0.962	0.963
TLI	0.955	0.956
RMSEA	0.059	0.060
Factor 1: Gait/Posture		
Speech	0.681	0.677
Facial expression	0.640	0.635
Arising from chair	0.752	0.781
Posture	0.800	0.788
Gait	0.744	0.761
Postural stability	0.572	0.591
Body bradykinesia and hypokinesia	0.874	0.861
Factor 2: Tremor		
Tremor at rest—face, lips, chin	0.621	0.604
Tremor at rest—right hand	0.220	0.236
Tremor at rest—left hand	0.772	0.752
Action/Postural tremor—right hand	0.480	0.499
Action/Postural tremor—left hand	0.818	0.833
Factor 3: Rigidity		
Rigidity—neck	0.471	0.461
Rigidity—right lower limb	0.914	0.917
Rigidity—left lower limb	0.892	0.882
Factor 4: Right		
Rigidity—right upper limb	0.682	0.687
Finger taps—right hand	0.594	0.601
Hand movements—right hand	0.712	0.709
Rapid movements—right hand	0.711	0.709
Leg agility—right leg	0.768	0.770
Factor 5: Left		
Rigidity—left upper limb	0.788	0.781
Finger taps—left hand	0.778	0.786
Hand movements—left hand	0.806	0.802
Rapid movements—left hand	0.814	0.798
Leg agility—left leg	0.797	0.726

CFI: Comparative fit index; TLI: Tucker-Lewis Index; RMSEA: Root mean square error of approximation.

**Table 3:** Descriptive statistics for UPDRS motor score and subscales (*n* = 193)*.

	Mean (SD)	Cronbach's alpha	Minimum observed value	Maximum observed value	Percent scoring possible minimum (best)/maximum (worst)*
Total Motor UPDRS Motor (27 items; range 0 to 108)					
OFF	25.0 (11.7)	0.89	5	66	0.0/0.0
ON	17.2 (10.8)	0.89	1	64	0.0/0.0
Gait/Posture (7 items; range 0 to 100)					
OFF	30.6 (17.2)	0.91	0	82	2.1/0.0
ON	23.5 (17.3)	0.91	0	82	3.6/0.0
Tremor (5 items; range 0 to 100)					
OFF	14.9 (12.6)	0.92	0	60	14.5/0.0
ON	8.8 (9.9)	0.92	0	55	33.7/0.0
Rigidity (3 items; range 0 to 100)					
OFF	14.8 (14.8)	0.91	0	58	27.9/0.0
ON	7.6 (11.5)	0.91	0	58	53.4/0.0
Right (5 items; range 0 to 100)					
OFF	27.7 (15.6)	0.91	0	80	3.6/0.0
ON	18.6 (14.4)	0.91	0	60	14.5/0.0
Left (5 items; range 0 to 100)					
OFF	29.0 (19.4)	0.91	0	80	9.4/0.0
ON	20.6 (16.9)	0.90	0	75	17.1/0.0

*All scales and subscales are scored so that higher values indicate more severely affected. Possible range for total motor UPDRS score is 0 (best) to 108 (worst). Possible range for subscales is 0 (best) to100 (worst).

**Table 4:** Pearson correlations between total UPDRS motor and subscales off and on medication.

	OFF medications						ON medications					
	Total motor UPDRS*	Gait/Posture	Tremor	Rigidity	Right	Left	Total motor UPDRS*	Gait/Posture	Tremor	Rigidity	Right	Left
OFF medications												
Total motor UPDRS*	1.00											
Gait/Posture	0.63^b^	1.00										
Tremor	0.19^a^	0.17^a^	1.00									
Rigidity	0.45^b^	0.35^b^	0.10	1.00								
Right	0.57^b^	0.52^b^	0.16^a^	0.40^b^	1.00							
Left	0.65^b^	0.61^b^	0.14^a^	0.45^b^	0.50^b^	1.00						
ON medications												
Total motor UPDRS*	0.90^b^	0.80^b^	0.36^b^	0.49^b^	0.64^b^	0.73^b^	1.00					
Gait/Posture	0.76^b^	0.90^b^	0.20^a^	0.30^b^	0.43^b^	0.56^b^	0.62^b^	1.00				
Tremor	0.32^b^	0.15^a^	0.81^b^	0.07	0.09	0.15^a^	0.27^b^	0.24^a^	1.00			
Rigidity	0.47^b^	0.32^b^	0.14	0.72^b^	0.34^b^	0.34^b^	0.43^b^	0.33^b^	0.13	1.00		
Right	0.69^b^	0.51^b^	0.17^a^	0.40^b^	0.83^b^	0.50^b^	0.59^b^	0.49^b^	0.17^a^	0.38^b^	1.00	
Left	0.80^b^	0.61^b^	0.20^a^	0.45^b^	0.52^b^	0.87^b^	0.70^b^	0.61^b^	0.23^a^	0.42^b^	0.59^b^	1.00

*Total UPDRS motor scores are adjusted for overlap with subscales. For example, correlation total UPDRS motor with Gait/Posture subscale was calculated as: Total UPDRS OFF with no Gait/Posture OFF items = Total UPDRS OFF − Gait/Posture OFF items.

^a^P < 0.05.

^b^P < 0.0001.

**Table:** (a)

How Parkinson's disease affects you on a day-to-day basis? (*n* = 170)						
	No difficulties (*n* = 27)	Mild difficulties (*n* = 94)	Moderate difficulties (*n* = 39)	High levels or extreme difficulties (*n* = 10)	*F* ratio*	Relative validity
OFF medications						
Total motor UPDRS	18.2^a^	23.3^a,b^	28.3^b^	42.4^c^	14.88	35.43
Gait/Posture	21.4^a^	27.4^a^	39.5^b^	53.9^c^	15.94	37.95
Tremor	13.3^a^	14.7^a^	13.6^a^	20.5^a^	0.95	2.26
Rigidity	7.1^a^	14.2^a^	15.0^a^	30.0^a^	6.58	15.67
Right	22.8^a^	26.3^a^	29.1^a^	46.5^b^	6.44	15.33
Left	19.3^a^	26.8^a,b^	33.3^b^	49.0^c^	7.48	17.81
ON medications						
Total motor UPDRS	11.6^a^	15.5^a,b^	20.5^b^	33.7^c^	15.73	37.43
Gait/Posture	15.2^a^	20.1^a^	33.1^b^	44.6^c^	15.70	37.38
Tremor	7.6^a^	8.8^a^	7.4^a^	10.5^a^	0.42**	1.00
Rigidity	2.5^a^	7.0^a^	7.7^a^	22.5^b^	8.49	20.21
Right	14.4^a^	17.2^a^	20.1^a^	36.0^b^	6.74	16.05
Left	12.6^a^	18.7^a,b^	23.1^b^	44.5^c^	10.76	25.62

All scales/subscales are scored so that higher values indicate more severely affected. Possible range for total motor UPDRS score is 0 (best) to 108 (worst). Possible range for subscales is 0 (best) to 100 (worst).

^a^, ^b^, and ^c^ means within a row with different letters differ significantly (*P* ≤ 0.05; Duncan multiple range).

*One way between group ANOVAs of total motor UPDRS or subscale and day-to-day effects of PD.

**Reference subscale = ON medication − Tremor.

**Table:** (b)

During the last 6 months, how would you rate the severity of your Parkinson's disease symptoms?						
	No symptoms (*n* = 4)	Mild symptoms (*n* = 79)	Moderate symptoms (*n* = 79)	Severe symptoms (*n* = 8)	*F* ratio*	Relative validity
OFF medication:						
Total motor UPDRS	14.0^a^	21.3^a,b^	27.6^b,c^	36.1^c^	8.28	5.56
Gait/Posture	16.1^a^	25.3^a,b^	35.6^b,c^	43.8^c^	7.70	5.17
Tremor	8.8^a^	12.9^a^	16.1^a^	18.8^a^	1.49**	1.00
Rigidity	12.5^a^	11.8^a^	15.9^a^	20.8^a^	1.64	1.10
Right	22.5^a^	25.3^a,b^	29.1^a,b^	38.1^b^	2.11	1.42
Left	8.8^a^	23.9^a,b^	32.0^b,c^	46.9^c^	6.57	4.41
ON medication:						
Total motor UPDRS	8.8^a^	14.1^a,b^	19.0^b^	31.1^c^	9.64	6.47
Gait/Posture	11.6^a^	19.2^a,b^	27.3^b,c^	39.7^c^	6.55	4.40
Tremor	1.3^a^	7.2^a,b^	9.4^a,b^	14.4^b^	2.66	1.79
Rigidity	6.3^a^	4.5^a^	9.7^a^	12.5^a^	3.39	2.28
Right	13.8^a^	16.7^a^	19.2^a,b^	31.9^b^	3.03	2.03
Left	8.8^a^	16.4^a^	22.2^a^	44.4^b^	8.67	5.82

All scales/subscales are scored so that higher values indicate more severely affected. Possible range for total motor UPDRS score is 0 (best) to 108 (worst). Possible range for subscales is 0 (best) to 100 (worst).

^a^, ^b^, and ^c^ means within a row with different letters differ significantly (*P* ≤ 0.05; Duncan multiple range).

*One way between group ANOVAs of total motor UPDRS or subscale and rating of severity of PD symptoms.

**Reference subscale = OFF medications − Tremor.
